# On the Climate of the Mountain Ranges of California in Relation to the Treatment of Phthisis

**Published:** 1862-07

**Authors:** James Blake


### ON THE CLIMATE OF THE MOUNTAIN RANGES OF CALIFORNIA, IN RELATION TO THE TREATMENT OF PHTHISIS.

By JAMES BLAKE, M. D.

In some former papers on Phthisis, published in this Journal, I have recommended the Coast Range of mountains as a preferable locality for the out-of-door residence of consumptive patients during the summer months. At the time I wrote these articles, I had no exact data as to the climate of this locality, my preference being founded on the beneficial influence that a camp life in these mountains had exerted in arresting the progress of the disease, in many of the patients I had sent there. At my request, one of my patients, who passed the last summer there, took the trouble to make a record of the temperature during July, August and a part of September, and the data I have thus obtained, furnish a sufficient explanation of the beneficial effects observed to follow a residence in these localities.

The following figures will enable the members of the profession to judge of the advantages of the climate for consumptive patients. They can, I am sure, be relied on, so far as they go, as I furnished the thermometers, one of which was suspended at the N. W. side of a tree, for the morning and noon temperature, the other on the N. E. side for the evening temperature, and the observations can be depended upon as correctly recorded. The hours at which the observations were taken, do not probably furnish the extreme temperature, as at least in July, the atmosphere has been somewhat warmed at 6 A. M., but the total variations are so slight, that the subtraction of a degree the 2 P. M. temperture, will probably afford a very close approximation to the truth. The locality of the camp was at a supposed elevation of 4,500 feet, about fifty miles north west of Colusa, and about twenty miles from the eastern foot hills of the Coast Range. For the sake of comparison, I have appended a statement of the mean temperature, and of the extremes, as observed at Iowa Hill a locality in the Sierra Nevada, at about the same elevation. The observations were not made at the same time, but in the year 1855. They, however, together with others, show that the climate of the Sierras very closely approximates to that of the Sacramento valley, and therefore it is far less favorable as a summer residence for consumptive patients than that of the Coast Range.

Observations of the temperature were taken three times a day, 6 A. M., 2 P. M., and 6 P. M. I have only, however, given the monthly average of these observations, the extreme daily range in the month, and the mean daily range; and also the extreme range observed during the month. These data afford sufficient elements to judge of the climate, as far as its temperature is concerned, and when compared with the figures obtained from observations made in the Sierra and the valleys, plainly show wherein its advantages consist.

COMPARISON OF TEMPERATURE, AS OBSERVED ON THE COAST RANGE, AND AT IOWA HILL, ON THE SIERRA, AT AN ELEVATION OF ABOUT 4,300 FEET.

*Mean Temperature for the Month.*

| | Max. | Min. | Mean. | |
|---|---|---|---|---|
| Coast Range, | 75.6 | 60.8 | 66.2 | (corrected) |
| Iowa Hill, | 90.3 | 63.7 | 77.0 | (observed) |

| | *Coast Range.* | *Iowa Hill.* |
|---|---|---|
| Mean diurnal variation, | 14.8 | 26.6 |
| Maximum temperature, | 82 | 97 |
| Minimum temperature, | 57 | 52 |
| Extreme variation, | 25 | 47 |
| Extreme variation on any day during the month, | 20 | 35 |

*August—Temperature at Camp on Coast Range.*

| | 6 a. m. | 2 p. m. | 6 p. m. |
|---|---|---|---|
| Mean temperature, | 55.3 | 69.5 | 63.7 |

Highest temperature during the month, 74; lowest, 47; mean daily variation, 14.2; extreme variation, 29; extreme variation on any one day, 18.

*September—First Fourteen Days.*

| | 6 a. m. | 2 p. m. | 6. p. m. |
|---|---|---|---|
| Mean temperature, | 51.5 | 65.0 | 57.0 |

Highest temperature during the month, 76; lowest, 45; mean diurnal variation, 13.5; extreme variation during the month, 31; extreme variation during any one day, 18.

The above figures show that, as regards temperature, our Coast Range, during the summer months, affords one of the most desirable localities for the treatment of our phthisical patients.

The temperature for July, is perhaps, that which is the most agreeable for living out in the open air—the thermometer never below 57 deg., and never higher than 82 deg., and with a variation, during the twenty-four hours, never greater than 20 deg. The comparison between the Sierra, at the same elevation, affords a striking contrast, for, whilst the average diurnal variation, in the first case, is only 14 deg., that of the Sierra is nearly twice as great, or 26.6 deg. An analogous contrast is evident through every important thermometric element of the two climates.

But it is not only in the advantages, as regards temperature, that the Coast Range affords a most suitable climate for phthisical patients, as in this respect it might be equalled, and perhaps surpassed, by many other localities, as for instance, the Sandwich Islands, Madeira, and other sea climates; but in this State, and, probably, in this State alone, are to be found combined all those climatic influences, which are even of greater importance than mere temperature, in the treatment of the disease.

Together with this agreeable temperature, the atmosphere is dry and bracing—almost always calm, and the sky is unclouded for months together. The last summer, there was no rain from the beginning of May until the end of November, so that my patients were not driven in by the weather until November. Towards the middle of September, they left their summer camp, for a lower point on the mountains, gradually descending as the season advanced. Unfortunately, I did not have any observations for other summer months, as it was not until they reached the greatest elevation, at the beginning of July, that observations were commenced. The climate, however, of the lower ranges, is analogous to that at greater elevations, and affords one of the most desirable localities for our patients, during the early summer and late autumn months.

Another important advantage the Coast Range of mountains possesses, is in the abundance of game, so that a good hunter can always keep the camp supplied.

With the return of winter, however, our patients necessarily have to seek the badly ventilated, sunless rooms of our habitations, a great deal of the good that has been done during the summer is apt to be lost. Should the case be not very far advanced, and the improvement during the residence in the mountains have been as great as usually takes place, with care, our patients can hold their ground, during an average winter, in our coast valleys, or on the lower hills of the Sierra. But where they can manage to leave, they can, by going a few hundred miles down the coast, again enjoy all the advantages afforded by their summer residence in the mountains.

In the northern parts of Mexico, the rainy season, which corresponds to our winter, sets in in June and July, and is over by the end of October or beginning of November; after which time, no rain falls until May or June. There are many points on the coast, where the mountain range of the Sierra, commences at a slight distance from the sea, and, although the low land is unhealthy, being subject to malarious diseases, yet as soon as a moderate elevation is attained, the climate becomes dry and bracing, and very analogous to that of our own Coast Range during the summer. Fortunately, these locali-
San Francisco and Mazatlan, is taking place, either by steamer or sailing vessels, three or four times a month. The country is well settled, and as large mining operations, conducted by our countrymen, are being carried on there, all sorts of supplies can be procured. About eighty miles back from the coast, the mountains already have an elevation of some thousand feet, and, although I have no accurate meteorological observations of the climate, yet, from the description of it by
ties are now easily reached, as the communication between
those who have passed the winter there, I am confident that it must be very analogous to that of our Coast Range. Our patients thus enjoy a perpetual summer, or, at least, a climate in which they can pass the whole of the year in the open air.

I believe, in no other part of the civilized world, can such favorable condition be found for treating phthisis, as on this coast. That the disease cannot be cured by drugs, is a fact becoming generally admitted by the profession. That it can be cured by out-door life in the mountain air, is a fact of which I have seen abundant proofs, and which others, who have crossed the Plains, have had opportunities of verifying. My own opinion is, that a large proportion of the cases of phthisis, will be found to yield to one or two years' open air treatment, and where the circumstances of the patient enable him fully to carry out such a plan, this coast offers, I think, by far the most favorable locality of any yet resorted to by phthisical patients—*Pacific Journal.*

